# Return to Work After Type A Aortic Dissection

**DOI:** 10.7759/cureus.100917

**Published:** 2026-01-06

**Authors:** Matthew J Billy, Salmaan Zafer, Christian Summa, Jason Stanton, Caroline K Chen, Zachary Brennan, Jiatian Qu, Tyler J Wallen

**Affiliations:** 1 General Surgery, Geisinger Health System, Scranton, USA; 2 Cardiac Surgery, Cedars Sinai Medical Center, Los Angeles, USA; 3 General Surgery, Geisinger Commonwealth School of Medicine, Scranton, USA; 4 Cardiovascular Surgery, Geisinger Health System, Wilkes-Barre, USA

**Keywords:** aortic dissection, lost income, missed work days, return to work, return-to-work, type a

## Abstract

Objective

Acute type A aortic dissection (TAAD) frequently affects patients during working years, but patient-centered employment outcomes after operative repair are not well characterized. The aim of this study was to describe return-to-work and self-reported income loss among 30-day survivors using a voluntary survey within a dual-center, single health system.

Methods

After Institutional Review Board (IRB) approval, we conducted a retrospective review of a prospectively maintained database and included any patient who suffered an acute type A aortic dissection that was treated operatively. We analyzed those who survived beyond 30 days. We subsequently contacted each of these patients to enroll them in a voluntary survey to assess their pre-operative and post-operative occupation, associated salary, and loss thereof from their recovery and perioperative period. Statistical analysis was then performed.

Results

A total of 173 patients who underwent urgent or emergent repair of TAAD from 2012 to 2023 were identified, and an attempt to contact each of them was made. Out of 173 patients surveyed, 49 were willing to participate in the survey. Out of 49 surveys collected, 22 were not filled out completely and were therefore excluded from the study, leaving 27 completed surveys. The mean number of missed working days as a result of undergoing surgical intervention was 103 days, and the average amount of direct missed income was $3347.22 per patient. Additionally, only 62.9% (17 out of 27) of patients returned to full- or part-time work after aortic surgery. In an exploratory stratification by annual income (<$40,000 vs ≥$40,000), return-to-work was two of six (33.3%) versus 15 of 21 (71.4%), respectively; this comparison was underpowered and not statistically significant.

Conclusions

Among survey respondents, prolonged time away from work after operative repair of TAAD was common, and a substantial proportion did not return to work. Because the respondent cohort was small and selected, these findings should be interpreted as descriptive and hypothesis-generating. Larger prospective studies are needed to evaluate predictors of return-to-work and to assess whether structured rehabilitation and survivorship support improve vocational recovery.

## Introduction

Ascending aortic dissection is a highly morbid condition that requires prompt surgical intervention [1-2]. This condition is characterized by the tearing of the inner aortic wall, creating a false plane and lumen that can result in distal ischemia [3]. Early and prompt recognition, management, and intervention are important in order to improve patient outcomes and mortality rates. This is often a large undertaking in the setting of a dangerous disease and, as a result, there is substantial mortality and morbidity associated with this pathology. Long-term outcomes from the International Registry of Acute Aortic Dissection (IRAD) suggest a 21% mortality in patients less than 70 years of age and 31% in those greater than or equal to 70 years of age (p=0.003). Of note, fewer elderly patients with type A aortic dissections (TAAD) were managed surgically than younger patients (71% versus 89%; p<0.001), with medical management alone having higher mortality rates regardless of age [4]. TAAD has a known massive economic burden and places a large strain on hospital systems, resources, and staff [5]. The economic impact has been well studied per patient, including re-admission cost, and these numbers have even been stratified by high- and low-volume centers [5]. Zhou et. al suggest that high-volume centers (defined as the top quartile of total repairs) had greater admission costs ($114,859 vs. $72,090, p<0.001) and similar readmission costs of upwards of $49,000 [5]. The data surrounding the economic burden primarily focuses on the cost to the system rather than the impact on the individual patient, which is poorly elucidated in the current literature.

Additionally, TAAD frequently affects patients in their working years, with data from IRAD suggesting the mean age to be 61.5 +/- 14.6 years, with 67.5% of these patients being male [4,6-7]. When looking at age as an independent factor, Howard et al. suggest that upwards of 35% of all aortic dissections occur in patients who are older than 75 [8]. This can be compared to the average age of patients undergoing coronary artery bypass graft (CABG) surgery, which has been increasing over the years, with data to suggest that more than 25% of all patients undergoing CABG are greater than 70 years of age [9]. This data further reinforces the fact that the majority of patients with acute cardiac pathology requiring intervention occur during the patients' working or economically productive years, and, therefore, the post-operative recovery can greatly impact their financial burden. 

The current literature focuses on the overall cost to hospital systems, large networks, or states. Although this is important to the system, it does not address the notable change both physically and economically that happens in the individual patient's life after suffering from a TAAD. This is an especially relevant consideration due to the incidence of TAAD occurring during patients' working years, in which they additionally may have several dependents [6]. An aspect that is not often addressed in the literature, but is also relevant when discussing outcomes, is the quality of life after surviving such a life-threatening pathology. One study addressing survivorship by Breel et al. found that for patients at a pre-operative MET score of 4-6 (defined as light housework, i.e., letting the dog out or walking slowly), only 41% were able to reach this level of functioning post-operatively [10]. Additionally, when surveyed, 51% of patients reported worse cognitive functioning, including 49% who had significant memory loss or were able to concentrate for less than ten minutes [10]. This study also noted that only 21% were able to resume their previous employment. These functional and cognitive sequelae may plausibly contribute to difficulty returning to prior employment, but employment outcomes remain incompletely characterized. All of this suggests that the ramifications of suffering from TAAD go beyond just the physical and mental stress that it causes, and there is a significant financial and social impact on these patients. The aim of this study is to focus on the socioeconomic burden to patients, their families, and their communities. This study specifically looks at how the patients in our community who suffered a TAAD and underwent an operation were affected in terms of their ability to return to work and the subsequent socioeconomic impact this had. Prior work has largely focused on clinical outcomes and hospital-level costs after TAAD. Less is known about patient-centered indirect consequences after survival, including return-to-work and financial disruption. Therefore, we conducted a retrospective review of a prospectively maintained aortic surgery database and administered a voluntary survey to describe employment status, time away from work, and self-reported income loss among 30-day survivors after operative repair of acute TAAD within a single health system.

This article was previously presented as a meeting abstract at the 2024 American Association for Thoracic Surgery Aortic Symposium, April 25-26, in New York City, New York. 

## Materials and methods

Study design

In this study, we conducted a dual-center, single-health system retrospective review designed to assess the socioeconomic impact of acute type A aortic dissections (TAAD) on our patients' ability to return to work, amongst other indirect costs. Institutional Review Board (IRB) approval was obtained prior to the commencement of our study design and study initiation. Data were drawn retrospectively from a prospectively maintained aortic surgery database and supplemented with patient-reported survey responses.

Inclusion and exclusion criteria

All patients in our database who experienced an acute TAAD between 2012 and 2023 and subsequently underwent an urgent or emergent operative repair were then eligible for inclusion. We only included patients who survived beyond 30 days post-operatively. Patients who declined participation in the survey or whose surveys were incomplete were excluded from the final analysis. Surveys were considered complete for analysis if responses were available for return-to-work status and time away from work (Q3 and Q5). Surveys missing these required outcome fields were excluded from the analytic cohort.

Data collection

A total of 173 patients were identified as meeting initial eligibility criteria across multiple hospitals within our health system. Each patient was contacted individually and invited to participate in a voluntary survey. The survey collected information on: preoperative employment status (working vs. not working), employment type (full-time vs. part-time), ability to return to work post-operatively, duration of time out of work, nature of return (full-time vs. part-time), and pre-dissection annual income, as well as self-reported loss of income attributable to the recovery period.

Eligible patients were contacted by telephone using the contact information listed in the electronic medical record. Calls were conducted using the standardized script (Appendix). Up to three contact attempts were made per patient on different days and times. Verbal consent was obtained prior to survey administration. Patients who could not be reached after attempted contact were considered nonresponders.

Statistical analysis

Descriptive statistics were calculated for baseline demographic and socioeconomic characteristics. Mean values were reported for missed workdays and self-reported income loss. Exploratory unadjusted comparisons between subgroups were performed, with categorical outcomes compared using Fisher's exact test and continuous outcomes compared using Student's t-test when appropriate. Given the small respondent sample size, analyses were interpreted as descriptive and exploratory. Statistical significance was defined as p<0.05. Analyses were performed in Microsoft Excel (Microsoft, Redmond, Washington).

## Results

**Figure 1:**
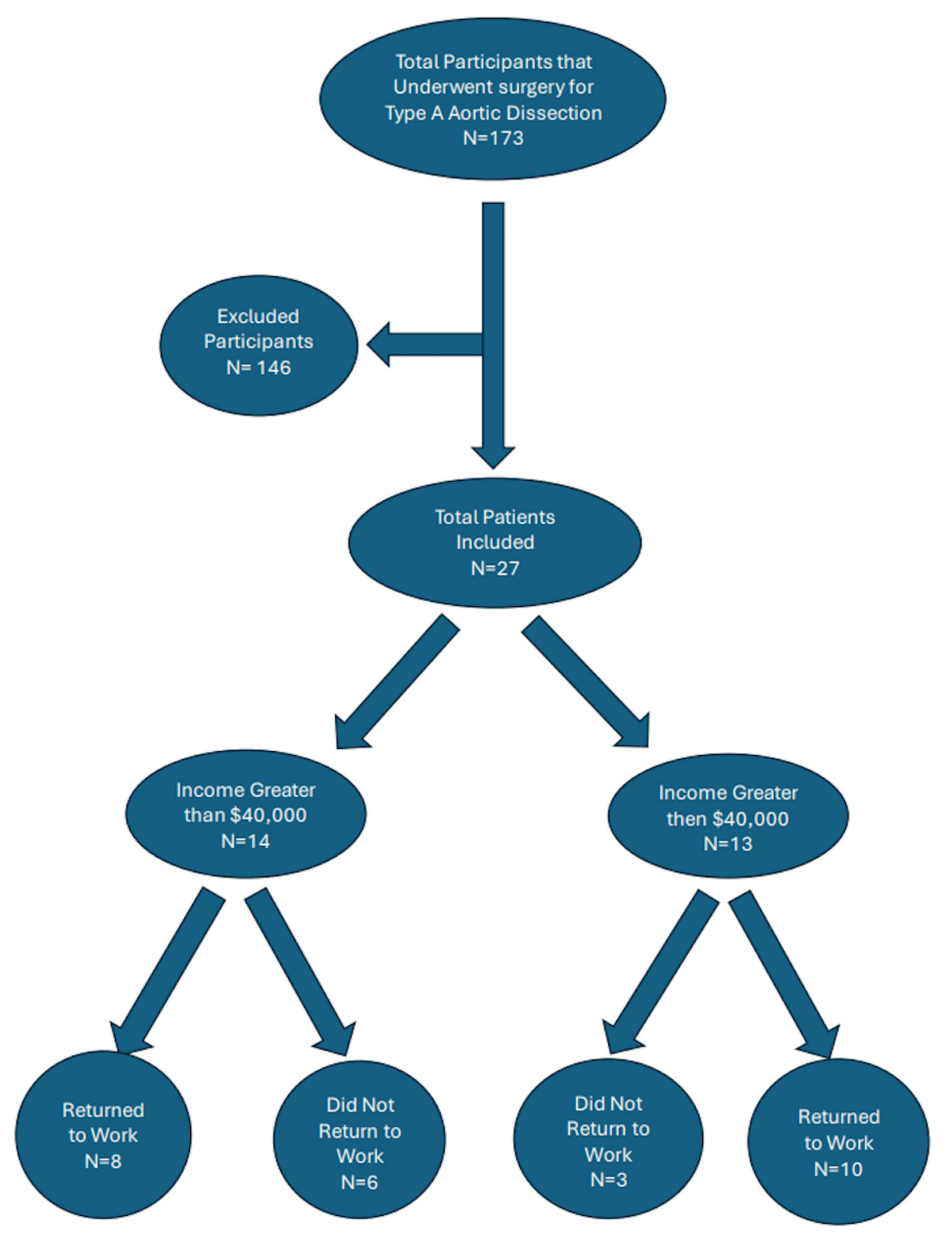
Flowsheet showing the breakdown of the individuals included in the study, further divided into income above and below $40,000 and the number of individuals who returned to work within their respective groups

We identified 173 patients who underwent urgent or emergent repair of TAAD from 2012 to 2023 across multiple hospitals within a single health system. Out of 173 patients that fit the criteria, 49 (28%) were willing to participate in the survey; however, only 27 (16%) were complete and therefore included in the study (Figure 1).

**Figure 2:**
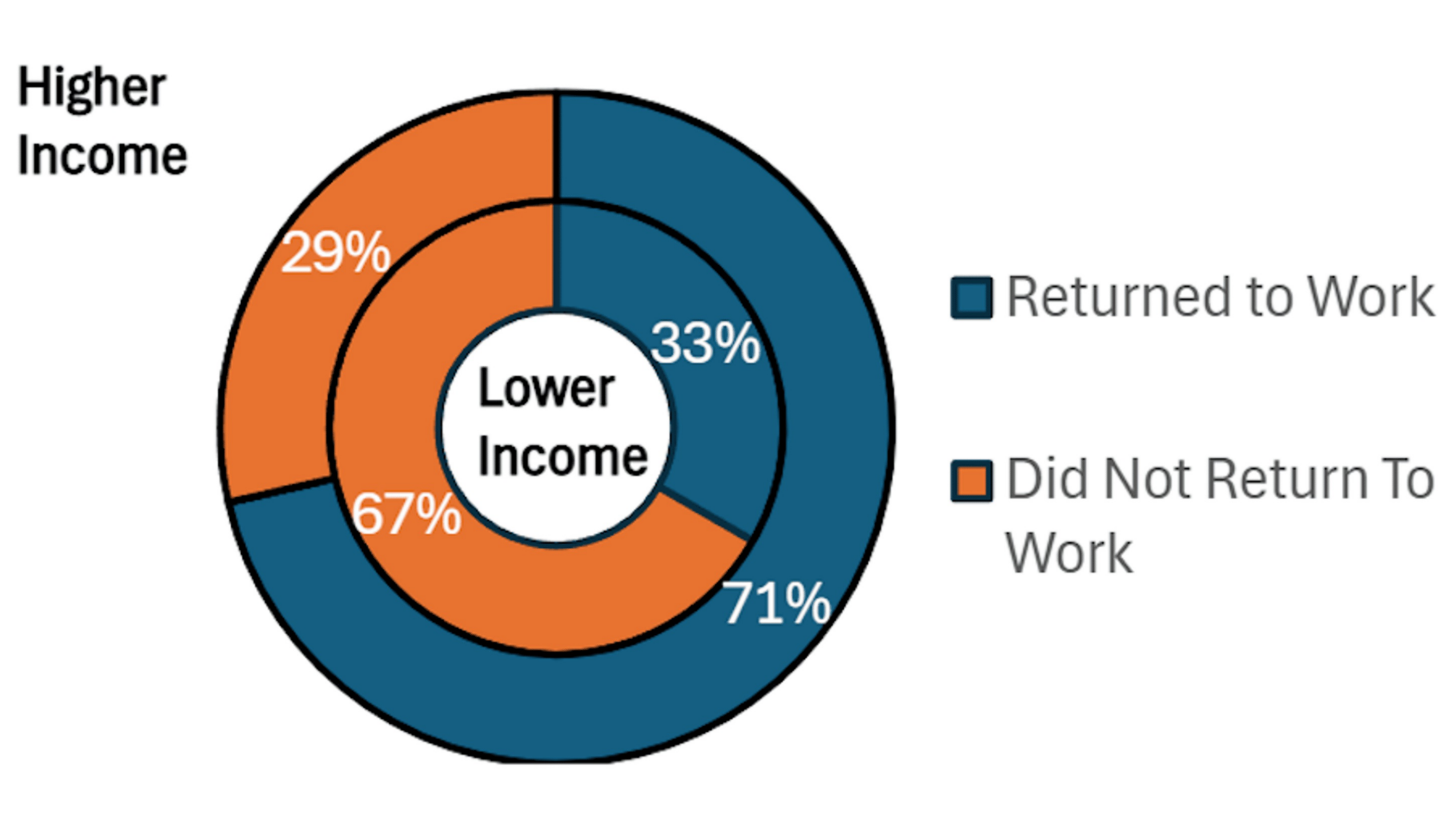
Percentage of individuals that return to work based on an income greater than or less than $40,000. Results shown are descriptive for survey respondents (n=27)

In terms of the number of individuals who returned to work, among respondents, 17 out of 27 (63%) reported returning to full- or part-time work after aortic surgery(Figure 2). We then stratified patients into individuals with income higher than or less than $40,000 per annum. Among respondents, there was a total of six lower-income patients, and only 33% of them had returned to work (two out of six). This was compared to 15 out of 21 (71%) of patients within the higher income group who returned to work. Though not statistically significant, more patients in the higher income group returned to work (0.714 vs 0.333; p=0.0883), suggesting an even larger financial burden on a group of people that may not have been financially secure prior to suffering a significant medical ailment event. However, it is important to note that subgroup comparisons are underpowered and exploratory, given small sample sizes and lack of statistical significance. 

**Figure 3:**
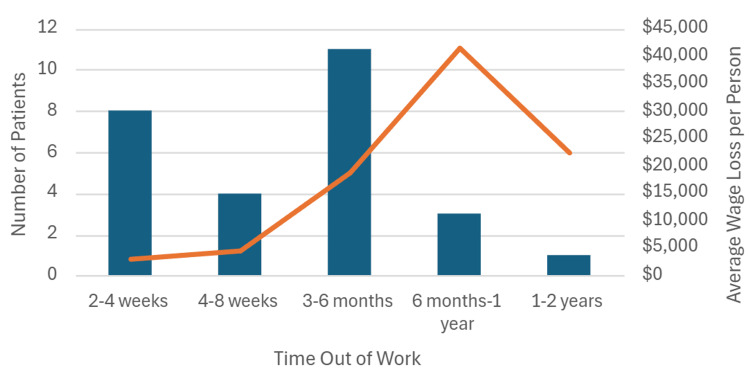
Average amount of work missed and resulting income lost for individuals requiring aortic surgery for TAAD. Results shown are descriptive for survey respondents (n=27). TAAD - type A aortic dissection

When analyzed for the amount of direct income lost per individual, the average was $3,347.22 per patient; however, some individuals had lost upwards of $42,000 after missing between six months and one year of work. Figure 3 represents the average wage lost per person as well as the amount of time out of work these individuals were required to take secondary to their recovery.

**Figure 4:**
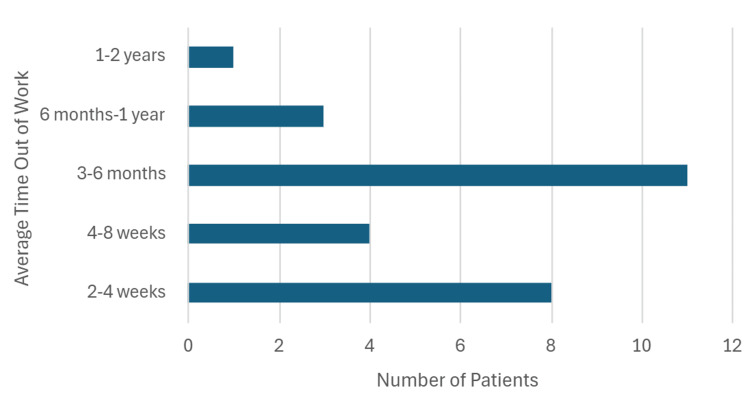
Average amount of work time missed after undergoing aortic surgery. Results shown are descriptive for survey respondents (n=27).

The overall mean number of missed workdays was 103 days. Individuals were grouped into either two to four weeks, one to two months, three to six months, six months to one year, or one to two years in terms of the number of missed workdays (Figure 4). Most often, individuals missed between three and six months of work (11 out of 27, or 40.1%). The total number of missed workdays from the participants of this study was 2790 days, amounting to a total of $395,892 of income lost as a result of having suffered a TAAD. This represents not only a significant economic hardship to the individual who suffered from a TAAD, but also the families they may have been supporting. These findings reflect outcomes reported by survey respondents and should be interpreted as descriptive, given the small, selected cohort.

## Discussion

Aortic dissection is a life-threatening condition caused by a tearing in the inner aortic wall, creating a false lumen. This false lumen is propagated by the blood entering between the intima and media. This compromise in the aortic wall can lead to malperfusion syndromes. Prompt recognition of symptoms is vital to pursue appropriate management, such as blood pressure control, pain management, and possible surgical intervention. Without appropriate care, dissections have significantly high mortality rates. As a result, the impact of TAAD and the associated morbidity and mortality has substantial consequences on the lives of the patients they afflict. Given the five-year survival rate after surgery is around 70-80%, the socioeconomic impact is an important short- and long-term consideration [11].

Part of the consideration for these patients is not just the impact of the surgery but also the long-term complications and recurrence risk. Even patients who undergo successful surgical treatment are still at long-term risk for re-dissection, aneurysm, myocardial infarction, or congestive heart failure. Approximately 20-30% require additional procedures within the first five years of the index operation.

Acute type A aortic dissection (TAAD) remains a highly morbid condition despite advances in technology, sensitivity of detection and diagnosis, and surgical management. Even with prompt identification and repair, many patients experience significant morbidity, need for re-operative intervention, and diminished quality of life. Our study and findings highlight an additional, less frequently studied dimension of survivorship - the socioeconomic burden of TAAD, specifically patients' and their ability to return to work, and missed opportunity costs. 

Socioeconomic impact of TAAD

We found that patients missed an average of 103 workdays following TAAD repair, with associated lost wages ranging from $3,347 to $42,000 per patient. Furthermore, only a portion of patients were able to return to work, and those in lower income brackets demonstrated a trend toward worse employment outcomes. These results are consistent with prior investigations showing that many survivors experience functional decline and difficulty resuming employment. For example, Breel et al. reported that only 21% of Dutch patients were able to return to their previous occupation after TAAD, with over half experiencing persistent cognitive impairment [10]. Similarly, Olsson et al. found that survivors of aortic dissection often require long-term medical follow-up and face reduced quality of life, further limiting employability [12]. By quantifying missed workdays and income loss, our study extends this literature by focusing specifically on indirect financial consequences rather than clinical or quality-of-life outcomes alone. These results should be interpreted as descriptive outcomes among survey respondents and are not intended to provide population-level estimates or causal inference.

Vulnerability of lower-income patients

The lower-income subgroup had a numerically lower return-to-work rate; however, this comparison was underpowered and not statistically significant, and should be viewed as hypothesis-generating (0.714 vs 0.333; p=0.0883). Unfortunately, we were not able to demonstrate a statistical difference between these two groups due to the small number of low-income respondents. However, this study further emphasizes the negative impact that TAAD has on the affected patients and their communities. This study shows that this impact is more substantial for patients in a lower-income bracket who are already at higher risk for financial hardship. This helps demonstrate that there needs to be a multi-disciplinary approach to assist patients returning to work and resuming the lives that they previously had before being affected by this pathology.

This echoes prior studies of cardiovascular surgery patients in general, which demonstrate that lower socioeconomic status is associated with poorer recovery, higher rates of depression, and reduced return-to-work potential [13]. Our results, therefore, underscore the importance of integrating socioeconomic considerations into survivorship care planning. A multidisciplinary approach, including social work, vocational rehabilitation, and mental health support, may help mitigate these disparities.

Indirect costs and community impact

The burden of TAAD extends beyond the patient. Missed workdays and reduced income not only affect individuals and families but also have implications for local communities and employers. While several studies have quantified the direct hospital costs of TAAD-reporting hospital charges exceeding $70,000 to $110,000 per admission - few have addressed indirect costs borne by patients and their families [5]. Our findings complement this body of work by emphasizing the "hidden" financial toll, which is not captured in hospital-based cost analyses. Incorporating both direct and indirect costs is essential for accurate health policy planning.

Limitations

This study has important limitations. First, survey participation was voluntary, and the final analytic cohort represented 27 of 173 eligible 30-day survivors, creating substantial risk of nonresponse selection bias and limiting generalizability. Respondents may differ from nonresponders in ways that relate to employment outcomes, including health status, functional recovery, or socioeconomic stability. Second, the survey relied on patient-reported time away from work and income loss, which introduces recall bias, particularly for patients whose index operation occurred years before survey administration. Third, incomplete surveys were excluded, which may further enrich the analytic cohort for patients more able or willing to complete the survey. Fourth, given the small sample size and heterogeneity in clinical course and occupation type, subgroup comparisons are underpowered and should be interpreted as exploratory. Finally, this study was performed within a single health system, which may limit external validity [4]. Given these limitations, this study is best interpreted as a pilot, hypothesis-generating assessment that supports the feasibility and importance of prospectively measuring vocational recovery after TAAD.

Future directions

Our future work will focus on prospective data collection to minimize recall bias, improve response rates, and allow for more detailed subgroup analysis. Comparative studies involving other major cardiac surgeries (e.g., coronary artery bypass grafting, valve repair) would provide insight into whether TAAD patients are uniquely vulnerable to long-term socioeconomic disruption. Additionally, integration with quality-of-life assessments, as previously performed by Howard et al. (2013) and Shan et al. (2013), may provide a more comprehensive understanding of how socioeconomic and clinical outcomes intersect in this high-risk population [8-9]. Interventions aimed at facilitating return to work, such as structured rehabilitation programs and employer-based accommodations, should be systematically evaluated.

## Conclusions

In this exploratory survey of respondents who survived operative repair of acute type A aortic dissection, many reported prolonged absences from employment, and some did not return to work. Differences observed between income strata were exploratory and not statistically significant in this small cohort. These findings support the need for prospective studies with standardized follow-up to identify predictors of return-to-work and to evaluate multidisciplinary approaches that may support vocational recovery.

## Appendices

**Table 1:** Acute Aortic Dissection and Rupture- Return to Work Phone Script and Survey

Section	Content
Introduction	Speaker: Hello, I am a resident/representative in the Geisinger Health System and I am contacting you today to ask you a few questions regarding your aortic dissection or rupture. We are conducting a study to better understand how long it takes patients to return to work after suffering from an aortic condition and would like your permission to ask you a few questions about this. Your answers will be kept confidential and de-identified. Do you consent to this survey?
Consent	If Yes, proceed with survey. If No, thank the participant and end call.
Q1	Were you working when you suffered your aortic dissection/rupture? A. Yes ; B. No
Q2	Were you working part time or full time? A. Part time ; B. Full time
Q3	Did you return to work after recovering from your operation? A. Yes ; B. No
Q4	If you were working full time at that time, did you return to full time or part time work? A. Full time ; B. Part time
Q5	Approximately how long were you out of work after returning home from the hospital after your dissection? A. 2–4 weeks ; B. 4–8 weeks ; C. 3–6 months ; D. 6 months to 1 year ; E. 1 to 2 years ; F. Greater than 2 years
Q6	Did anyone else in your household have to take time away from work to assist in your recovery? A. Yes ; B. No
Q7	If yes, how long were they out of work? A. 2–4 weeks ; B. 4–8 weeks ; C. 3–6 months ; D. 6 months to 1 year ; E. 1 to 2 years ; F. Greater than 2 years
Q8	At the time of your dissection, what was your approximate yearly income? A. $10,000 or less ; B. $10,000 to $20,000 ; C. $20,000 to $40,000 ; D. $40,000 to $60,000 ; E. $60,000 to $80,000 ; F. $80,000 to $100,000 ; G. $100,000 to $120,000 ; H. $120,000 to $140,000 ; I. Greater than $140,000 ; J. Prefer not to share
Q9	If a caregiver took off work to assist in your recovery, what was their approximate yearly income at that time? A. $10,000 or less ; B. $10,000 to $20,000 ; C. $20,000 to $40,000 ; D. $40,000 to $60,000 ; E. $60,000 to $80,000 ; F. $80,000 to $100,000 ; G. $100,000 to $120,000 ; H. $120,000 to $140,000 ; I. Greater than $140,000 ; J. I don’t know
Closing	This concludes our survey. Thank you for participating in this survey.
